# Disposing of the Dead

**Published:** 1875-02

**Authors:** 


### DISPOSING OF THE DEAD.

An old burying-ground in England, which had been used for several hundred years, was abandoned, and its black, rich soil was sold to farmers to fertilize their gardens. This loam, enriched by the decomposition of multitudes of human bodies, wrought wonderful effects. The vegetables propagated under its influence grew rank and big and thrifty; the ornamental plants exhibited the greenest of green leaves and the most gorgeous floral hues, and all inanimate life took on new glory and more gorgeous beauty. It was evident that the earth contained some principle adequate to give vigorous life and impart infinite beauty, to all manner of vegetation.

But presently another effect was noticed. People were mysteriously taken sick. Typhoid fever became common, in a locality where it had never been known. Deaths occurred in increasing numbers. Within one year, more than one-tenth of the inhabitants of the immediate locality in which the powerful fertilizer was employed, were dead. Nobody now doubts the cause of this great mortality. It was surely due to the disturbance and the distribution of the earth of that old burying-place. It was evidently a dense, moist soil originally, a soil which held, rather than exhaled, the obnoxious gases resulting from decomposi tion. Had the soil been sandy, these poisonous emenations would have escaped long before; but not, perhaps, without destroying the lives of some of those exposed to the deleterious influences. If wells of water had been near at hand, they, too, would of necessity have been contaminated. One thing is clear; if our dead are to be buried in the ground, the place of their interment should be far away from the habitations of the living. There is poison enough in one dead and decomposing body to kill thousands of men and women. The danger is perpetuated by our system of burial. We prolong the period of decomposition by our carefully sealed coffins and caskets. We drop tears over the remains of our dear ones, who in life would not have harmed us for the world, and we neglect to observe that by our mode of giving "dust to dust," we have forged a weapon potent for evil.

It is now urged that we should bury our dead in wicker coffins, or baskets or network of some kind, so that the antiseptic and depurative power of earth may be more promptly exercised. This was the universal practice in ancient times among those who disposed of their dead by depositing them in the earth. It has its disadvantages, in a sanitary point of view. If the soil employed were loose and porous, it is clear that the noxious gases would readily escape to contaminate the air in the neighborhood; if the soil were moist and heavy, it would in effect seal up the emenations, so that their escape would be protracted over a long period, and the end sought would not be attained. The object of burial unquestionably is to provide a means whereby the body may, without offence to the living, be resolved into its original elements. Why coffins were ever introduced is a question. It could not have been in order to protect the lifeless form from contact with the earth; it must have been because it was discovered that in loose soils of sand and gravel, the early and offensive stages of decomposition proceeded at too rapid a rate for the well-being of the living.

There can be no doubt that the average duration of human life is shortened by our system of disposing of our dead. Small-pox has been communicated by handling ten years later the remains of a person who died with that malady. There is not a contagion known which has not been extended in a similar manner. There is not a burying-place in the vicinity of the residence of human beings, which has not accomplished something of its deadly work.

What then shall we do with our dead? The question is as important as it is delicate. We have no right to say that it shall not be discussed reverently and in a right spirit. It must not only be discussed, but the time is not far distant when a radical change must be made in our methods. We cannot go on for ever in the work of multiplying burials in the same spots. The soil cannot purify beyond a reasonable limit.

Some of the Indians of the Plains, who are wonderfully wise in many things, dispose their dead on scaffolds twenty feet high, covering them lightly with skins, the edges of which they weight with stones so that the fierce winds cannot disturb them. We have passed by these elevated graves at all stages, but have never detected the least odor from them. They contaminate only the upper air, which is not needed by anybody.

It would seem probable that we shall come to adopt burning, "cremation," in time. This process resolves the body to its first elements, and by a process at once innocuous and speedy. Nothing remains but a handful of wholesome ashes. No water-courses are poisoned, no destructive exhalations are thrown off. The gases of combustion escape, and are at once diluted with the vast air-supply overhead. With the combustion of the body all disease-germs would be forever consumed.

This result can only be attained by the exercise of reason and reflection. There is no human being so base, let us believe, as to be willing to destroy the lives of his fellow-beings after his demise. There are few who would not beg to be spared the dying reflection that their decomposing remains might carry death to some dear one for whose comfort they had labored in life, or might lessen the chances of living, for some human being.

When it comes to the point, we must settle this question, each for himself or herself. The fashion of to-day in civilized lands, is placing in the earth. It does not follow that the fashion is not a very bad one. It is certain that the fashion is a very dangerous one, and that each year multiplies its dangers. It is true that more people can live on the earth, in a given space, than can be buried in it with safety to those who are upon it. In view of all the facts, shall we not do wisely and well if we ask our friends not to give our bodies to prolonged corruption—a corruption which may carry disease and death to others in a thousand unseen ways—but that they dispose of whatever remains after our life-work is done, speedily and with a thoughtfulness so far-reaching that it shall include all humanity in its kindly embrace. If this can best be done by burning, then let that process be employed. But we live in the hope that some chemical means will ere long be suggested, by which complete solution will be secured without the necessity of the consuming fire. We shall be glad to give a hearing in our pages to any of our friends who may have ideas on this subject.

---

Don't deceive yourself by supposing that because your room is cold it is ventilated. The mercury may be at zero and yet the air may be too stale and exhausted to support life.

